# Application of CRISPR/Cas9 editing and digital droplet PCR in human iPSCs to generate novel knock-in reporter lines to visualize dopaminergic neurons

**DOI:** 10.1016/j.scr.2019.101656

**Published:** 2019-12

**Authors:** Christa Überbacher, Julia Obergasteiger, Mattia Volta, Serena Venezia, Stefan Müller, Isabella Pesce, Sara Pizzi, Giulia Lamonaca, Anne Picard, Giada Cattelan, Giorgio Malpeli, Michele Zoli, Dayne Beccano-Kelly, Rowan Flynn, Richard Wade-Martins, Peter P. Pramstaller, Andrew A. Hicks, Sally A. Cowley, Corrado Corti

**Affiliations:** a Institute for Biomedicine, Eurac Research, Affiliated Institute of the University of Lübeck, Bolzano, Italy; b Department of Biomedical, Metabolic and Neural Sciences, Università di Modena e Reggio Emilia, Modena, Italy; c Institute of Human Genetics, Munich University Hospital, Ludwig-Maximilians University, Munich, Germany; d CIBIO – Centre for Integrative Biology, Università degli Studi di Trento, Trento, Italy; e Department of Surgical Sciences, Dentistry, Gynecology and Pediatrics, Section of Surgery, University of Verona, Verona, Italy; f Department of Diagnostics and Public Health, University of Verona, Verona, Italy; g Center for Neuroscience and Neurotechnology, University of Modena and Reggio Emilia, Modena, Italy; h Department of Physiology, Anatomy and Genetics, University of Oxford, Oxford, UK; i Oxford Parkinson's Disease Centre, University of Oxford, Oxford, UK; j James Martin Stem Cell Facility, Sir William Dunn School of Pathology, University of Oxford, Oxford, UK

**Keywords:** Human induced pluripotent stem cells, Dopaminergic neurons, CRISPR/Cas9, Knock-in, Digital droplet PCR, Fluorescent reporter, FACS

## Abstract

Human induced pluripotent stem cells (hiPSCs) have become indispensable for disease modelling. They are an important resource to access patient cells harbouring disease-causing mutations. Derivation of midbrain dopaminergic (DAergic) neurons from hiPSCs of PD patients represents the only option to model physiological processes in a cell type that is not otherwise accessible from human patients. However, differentiation does not produce a homogenous population of DA neurons and contaminant cell types may interfere with the readout of the in vitro system. Here, we use CRISPR/Cas9 to generate novel knock-in reporter lines for DA neurons, engineered with an endogenous fluorescent tyrosine hydroxylase – enhanced green fluorescent protein (TH-eGFP) reporter. We present a reproducible knock-in strategy combined with a highly specific homologous directed repair (HDR) screening approach using digital droplet PCR (ddPCR). The knock-in cell lines that we created show a functioning fluorescent reporter system for DA neurons that are identifiable by flow cytometry.

## Highlights

• ddPCR is a highly sensitive and accurate screening tool for CRISPR/Cas9 engineering.
• Dopaminergic differentiation results in faithful co-expression of TH and reporter.
• Endogenous fluorescence detectable in dopaminergic neurons by flow cytometry.

## 1 Introduction

Parkinson´s disease (PD) is the second most common neurodegenerative disorder after Alzheimer's disease, and the most common neurodegenerative movement disorder (Nussbaum and Ellis 2003). It is characterised by resting tremor, rigidity, bradykinesia and postural instability. Symptoms of PD are associated with a profound loss of DA neurons in the substantia nigra pars compacta (SNpc) resulting in depletion of DA in the striatum and other projection areas. The details of the selective vulnerability of the DA neurons to neurodegeneration in PD are still unclear, despite several hypotheses having been proposed throughout the years (Bolam and Pissadaki, 2012). Midbrain DA neurons share the molecular marker protein tyrosine hydroxylase (TH), the rate limiting enzyme in biosynthesis of DA (Nagatsu et al., 1964). TH is commonly used as a marker to specifically identify DA neurons (Chambers et al., 2009; Fedele et al., 2017; Kirkeby et al., 2012).

Human induced pluripotent stem cells (hiPSCs) are an important resource for disease modelling of PD, especially because they are easily accessible, can be derived from PD patients and efficiently differentiated into functional DA neurons (Hartfield et al., 2014; Kirkeby et al., 2012; Kriks et al., 2011). Efficiency of both hiPSC derivation and the generation of DA neurons is variable from patient to patient and individual cell lines respectively. The most common approaches involve dual SMAD inhibition of cells cultivated either in a monolayer (Kriks et al., 2011) or as embryoid bodies (Kirkeby et al., 2012). There is, however, a lack of consistency in the literature on the yields of TH positive cells obtained through these methods and generally, a heterogeneous population of neurons can be observed. Both are important considerations to be taken into account when using this cell model, as depending on the readout method, it is not possible to exclude non-DA cells from analyses or to morphologically distinguish them from other neuronal cell types. Different reporter systems for TH have been described (Cui et al., 2016; Kelly et al., 2006) but only a recent study has shown that a fluorescent reporter driven by an endogenous promoter can faithfully mimic TH expression in vitro (Xia et al., 2017). Previous reporters were, however, created in human embryonic stem cells (hESCs) using transcription activator-like effector nucleases (TALENs), a technology for which not all laboratories have the capacities and expertise to produce.

Here, using a CRISPR/Cas9 genetic engineering approach, we report the generation of novel knock-in cell lines that carry an endogenous enhanced green fluorescent protein (eGFP) reporter for TH. We present an easily reproducible approach for CRISPR/Cas9 gene editing of hiPSCs, which we combined with a novel digital droplet PCR (ddPCR) assay design as a highly specific screening method for homology directed repair (HDR), thus facilitating the process. The ddPCR technique is based on partitioning of DNA and PCR mix into nanoliter-sized oil droplets, and allows absolute quantification of nucleic acids even of very low abundance (Hindson et al., 2011). We demonstrate that CRISPR/Cas9 allows a seamless knock-in and the production of reporter cell lines that allow identification of TH-expressing cells by Fluorescent – activated cell sorting (FACS).

## 2 Methods and materials

### 2.1 Cell lines

The genetic engineering was carried out on three hiPSC lines in parallel, obtained from healthy donors. Namely:

- 802#7
- SFC856-03-04 (STBCi063-A)
- SFC840-03-05 (STBCi026-C)

Both SFC856-03-04 and SFC840-03-05 are cell lines from the StemBANCC consortium, are listed in hPSC Reg https://hpscreg.eu/ and available through EBiSC https://ebisc.org/. They are derived from skin fibroblasts of healthy donors. The reprogramming method was non-integrating with Sendai virus (Cytotune Reprogramming Kit, Thermo Fisher, cat# A16517and A13780-01 respectively).

Cell line 802#7 was derived from skin fibroblasts collected at the University of Lübeck. Reprogramming method was non-integrating using episomal vectors.

**Table:** 

Cell line	Sex	Clinical status
802#7	F	healthy
SFC856-03-04 (STBCi063-A)	F	healthy
SFC840-03-05 (STBCi026-C)	F	healthy

hiPSCs were maintained in StemMACS^TM^ iPS-Brew XF with supplement (Miltenyi, cat #130-104-368) on Matrigel coated plates (Corning, cat# 354277) and EDTA is used for routine passage (Beers et al., 2012).

For testing in silico designed sgRNAs we used HEK 293T cells expressing *S. pyogenes* Cas9 under control of a Tetracycline inducible promoter.

### 2.2 GuideRNA design and testing by T7-Endonuclease assay

The sgRNAs were designed in silico via the CRISPR Design Tool http://crispr.mit.edu/. A total of four sgRNAs were selected and tested in vitro by T7 Endonuclease Assay. All sgRNAs were designed in order to target the region of the stop codon of the TH-gene. NGG triplets around the stop codon where specifically selected for the design.

Four different sgRNAs were tested (see key resource table for sequence). The sgRNAs were cloned into a LV-FU-U6-sg-bb vector.

300,000 HEK 293T Cas9 cells were plated for transfection and Cas9 expression induced with 10 µg/ml Tetracycline. The following day, cells were transfected with 1 µg sgRNA-carrying plasmid using Lipofectamine LTX reagent (ThermoFisher, cat# 15338100).

Cells were kept in the same media for at least 72 h. Then cells were lysed for gDNA extraction with QIAamp® DNA Blood Mini Kit (Qiagen, cat# 51104).

The region around the cutting site of Cas9 was amplified with Q5® High-Fidelity polymerase (New England Biolabs, M0492S). Primers were designed 400 bp upstream and 1000 bp downstream of the cutting site.

Primers

Fw (5′−3′) GGCTTAGGGATATGGTCAAGG

Rv (5′−3′) TGTTGGGTGCTCTCTCTGGA

For T7 Endonuclease assay, 200 ng of purified PCR reaction was used for heteroduplex formation.

Heteroduplex formation in the Thermocycler using the following conditions.

**Table:** 

Initial Denaturation	95 °C		5 min
Annealing	95–85 °C	−2 °C/second	
	85–25 °C	−0.1 °C/s	
Hold	4 °C		∞

To the annealed product, 1 µl of T7 Endonuclease (New England Biolabs, BM0302L) was added and the mixture was incubated for 15 min at 37 °C.

Reaction was stopped by adding 1.5 µl of 0.25 M EDTA (ThermoFisher, cat# 15575020).

To analyse the fragmented PCR the whole reaction was loaded onto a 2% Agarose (BioRad, 1613101) gel with a loading buffer without bromophenol blue. For DNA visualization GelStar™ Nucleic Acid Gel Stain (Lonza, LO50535) was used.

The gel image was acquired using a ChemiDoc™ Imaging Systems and analysed using ImageLab to measure the integrated intensity of not cleaved and cleaved bands (see Fig S1A)

For each lane the fraction of the PCR product cleaved (fcut) was calculated using the following formula (Ran et al., 2013): fcut = (*b *+ *c*)/(*a *+ *b *+ *c*), where a is the integrated intensity of the undigested PCR product and b and c are the integrated intensities of each cleavage product.

The percentage of Indel formation was estimated with this formula:

$$\text{Indel}\%=100\;\;\times (1-\sqrt{\left(1-\text{fcut}\right)})$$

sgRNA4 was identified as the most efficient. Sequence is: 5′ GACGCCGTGCACCTAGCCAA 3′, protospacer adjacent motif (PAM) sequence is TGG. The oligonucleotides for this sgRNA were cloned into the expression vector pX459V2.0-eSpCas9(1.1) (Addgene #108292) (Ran et al., 2013).

### 2.3 Plasmid construction for HDR

The donor sequence was cloned into the commercially available Zero Blunt™ TOPO™ PCR Cloning vector (ThermoFisher, cat# 450031). For homology arms, the sequences for left and right arm were amplified from genomic DNA (see key resource table for primer sequence), annealed to each other and subsequently inserted bluntly into the vector. T2A, eGFP, loxP-EF1a-Blast-loxP was cloned into the vector between the two 800 bp homology arms into XbaI (New England Biolabs, cat# R0145S) and XhoI (New England Biolabs, cat# R0146S) restriction sites (see supplementary information for FASTA sequence of TH Donor). The Stop codon of TH gene was in this way cut out of the sequence.

### 2.4 Electroporation of hiPSCs

For gene editing at the TH locus, a Neon electroporation system was used (ThermoFisher, cat# MPK5000), using methodology adapted for gene editing hiPSC (Buchrieser et al., 2017; Flynn et al., 2015). 3 × 10^6^ hiPSCs of each cell line were transfected by electroporation (1250 V, 20 ms pulse width, 1pulse) in a 100µL tip with 15 µg total DNA (3.11 µg sgRNA plasmid and 11.35 Donor plasmid). All plasmids used for electroporation were prepared using an endotoxin free MidiPrep Kit (ThermoFisher, cat# K210004). After electroporation, the cells were plated at high density (4 × 10^5^ cells/cm^2^) in StemMACS medium without Pen/Strep containing 10 µM Rock-Inhibitor (Miltenyi, cat# 130–104–169). 24 h after electroporation Puromycin (SantaCruz Biotechnology, cat# sc-108071A) selection was started with 0.5 µg/ml for 48 h. After another 24 h without Puromycin cells were replated onto MEF cells for clonal selection (see supplementary information) and some were lysed for gDNA extraction for HDR screen.

### 2.5 Digital droplet PCR homologous directed repair screening

**Fig. 1:**
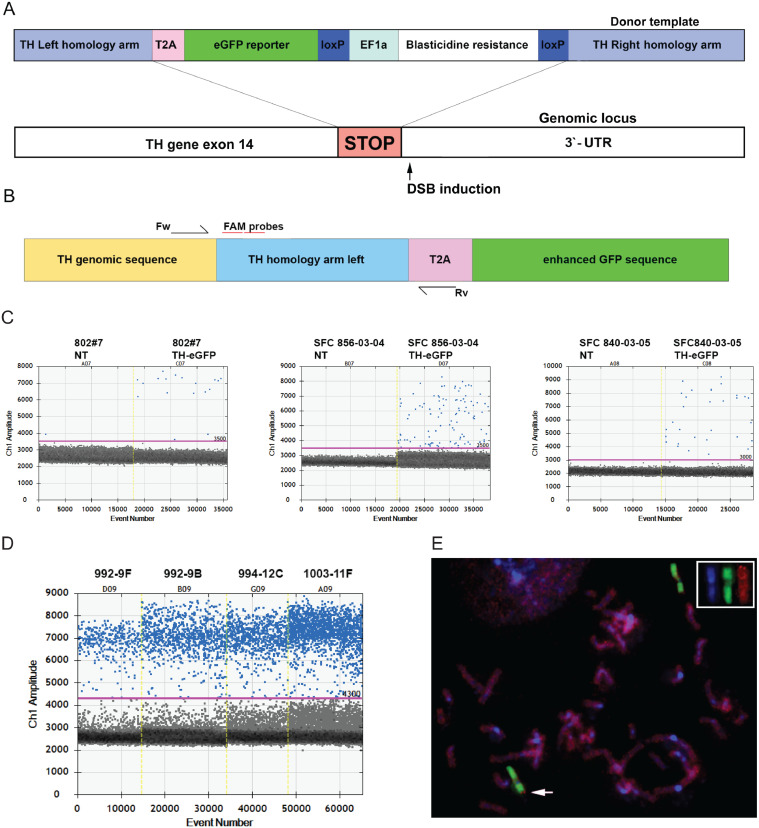
Generation of hiPSC knock-in lines with CRISPR/Cas9. (A) Schematic representation of the donor construct for homologous recombination at human TH locus. (B) Scheme of primer-probe position for ddPCR HDR screen (DSB, double strand break). (C) Raw droplet data of ddPCR measured for three different cell lines four days after electroporation with CRISPR plasmids. For each graph, a non-transfected control sample is shown on the left side, positive events detected for HDR sequence on the right side. Yellow lines indicate separation between sample reads. Blue dots represent positive droplets. (D) Raw droplet data of ddPCR measured for four cell clones selected showing dramatic increase in positive droplet count for HDR sequence after single clone selection. Yellow lines indicate separation between sample reads. Blue dots represent positive droplets. 992-9F and 992-9B are derived from 802#7 parental line, 994-12C from SFC 856-03-04 and 1003-11F from SFC 840-03-04. (E) Representative FISH image after in situ hybridization of the plasmid cloned TH donor construct (red) together with a chromosome 11 specific paint probe (green) to metaphase chromosomes (blue) of the successfully engineered clone 992-9B. Arrow points to the TH hybridization signal near the terminal end of the short arm of chromosome 11 (11p15.3), the chromosomal position of human TH gene (inset: display of split colour channels of the chromosome 11, highlighting the transgene insert).

For homologous directed repair (HDR) screening, gDNA from cells 96 h after electroporation was analysed. gDNA was extracted with QIAamp® DNA Blood Mini Kit (Qiagen, cat# 51104). Two hydrolysis probes were used for HDR detection (FAM) (Fig 1) and multiplexed with a standard reference gene hydrolysis probe (RPP30, HEX, BioRad cat# 10031243). For reaction setup, ddPCR Supermix for probes was used (BioRad, cat# 11969064001). For droplet formation Droplet Generation Oil for probes was used (BioRad, cat# 1863005). Droplet formation and PCR readout was carried out in BioRad systems for ddPCR.

For HDR screening, 130 ng of gDNA were used per reaction, equivalent to ca. 40000 DNA copies (one human genome = 3.3pg).

The assays used for HDR screen were made of the same forward and reverse sequence but had different probe sequences (probe 1 and 2, see key resource table).

Reaction was set up as followed.

**Table:** 

RPP30	1.1µl
Assay 1	1.1µl
Assay 2	1.1µl
gDNA (130 ng)	Variable
ddPCR™ Supermix for Probes (No dUTP)	11µl
H2O	Up to 22µl

A positive control was included in the beginning to assess assay quality. Positive control were non-transfected samples spiked with custom gBlocks® (double stranded synthetic DNA fragments) from IDT® identical to the sequence to be amplified from genomic DNA. gBlocks were used at a 10000-fold dilution from stock and gDNA from non-transfected samples at the same concentrations as transfected samples. Droplet formation was performed as indicated by manufacturer.

PCR run

**Table:** 

After PCR amplification, the plate was analysed in the QX200 Droplet Reader. Experiment Type was “ABS” (absolute quantitation) and fluorescence was read in FAM/HEX channels. Data was analysed using QuantaSoftTM software from BioRad. Amplitude threshold in Channel 1 and 2 was set individually for each channel and each probe combination. Also, amplitude threshold variation was possible between separately run plates.

After HDR screen, iPSCs were plated for clonal selection and screened for signal enrichment (see supplementary information for procedures). Identified clones were expanded and underwent further quality check by sequencing before continuing with Fluorescent-in-situ-hybridization.

### 2.6 Fluorescent-in-situ-hybridization (FISH)

For TH-FISH probe construction, an additional 2000 bp sequence homologue to the genomic sequence of TH was cloned upstream of the left homology arm, resulting in a total of 5000 bp of hybridization sequence. The TH-FISH probe was labelled with Cy3-dUTP (GE Healthcare, cat# PA13101) by nick-translation (Muller et al., 2007).

**Table:** 

Plasmid probe (2 µg)	Variable
10x Polymerase I Buffer	5µl
DNase I	1µl
DNA Polymerase I	1µl
dNTP mix without dTTP (0.2 mM each of dATP, dCTP, dGTP)	5µl
100 µM Cy3® dUTP Mix	2µl
H2O	To 50µl

Reaction was incubated for 90 min at 15 °C

To facilitate identification of the target chromosome, in some FISH experiments the TH-Plasmid was co-hybridized with a DOP-PCR labelled chromosome 11 specific painting probe (Muller et al., 2007).

Per FISH experiment, 500 ng Cy3-labeled TH-plasmid probe DNA, 200 ng Alexa488-labeled chromosome 11 paint probe, 1 µg salmon sperm DNA (ThermoFisher, cat# 15632011) and 10 µg human cot-1 DNA (Roche, cat# 11581074001) were mixed and ethanol precipitated for 30 min at −20 °C. The mix was centrifuged at 30,000 g for 15 min and then resuspended in 1.5 µl H20 and 3.5 µl LSI hybridization buffer (Abbott Molecular, cat# 30-804826).

To obtain chromosome preparations for FISH, cultured cells were incubated in Colcemide (Gibco, cat# 15210-040) solution for 4 h, followed by hypotonic treatment in 0.075 M KCl for 20 min at 37 °C, fixation with Carnoy solution (3:1 v/v methanol/acetic acid) and spreading on a wet microscopic slide with a plastic transfer pipette. After air-drying, the slide with the chromosome preparation was dehydrated in ascending ethanol concentrations (80%, 90% and 100%) for 3 min each, followed by incubation overnight on a heating plate at 50 °C.

Prior to hybridization, the FISH probes were denatured at 75 °C for 7 min. The probes were then pipetted onto the microscopic slide containing the metaphase spreads, covered with a glass cover slip and sealed using rubber cement. Slide denaturation and hybridization were carried out in a programmable temperature controlled slide processing system (HYBrite, Abbott Molecular) at 75 °C for 2 min and at 37 °C ON, respectively. Next, the cover slip was removed and slides were incubated at 75 °C in 0.4X SSC Tween®20 0.3% (pH 7) for 2 min. After that, they were washed in 4X SSC Tween®20 0.1% (pH 7) and finally mounted in Vectashield mounting medium with DAPI (Vector Laboratories, cat# H-1200) under a cover slip. Fluorescence microscopy, image capture and analysis were performed using a Zeiss© Axioplan2 epifluorescence microscope controlled by an ISIS FISH Imaging System (Metasystems).

### 2.7 In vitro differentiation of DA neurons

For differentiation according to (Zhang et al., 2014), cells were plated on day 2 on Matrigel coated dishes. hiPSCs were detached with TrypLE as described earlier and plated as a single cell suspension at a density of 35,000 cells/cm^2^ in StemMACS medium with 10 µM Rock-Inhibitor Y-27632. 24 h after plating, confluency was assessed microscopically. Differentiation was started at a confluency of about 50%, which was noted as Day 0.

For the differentiation, three media were needed. The media composition is listed in Table 1.

**Table. 1:** Media for dopaminergic differentiation.

Medium N1 100ml		Medium N2 100ml		Medium NB-B27 100ml	
DMEM, high glucose, GlutaMAX™ (ThermoFisher, cat# 31966021)	83ml	DMEM, high glucose, GlutaMAX™	98ml	Neurobasal (ThermoFisher, cat# 21103049)	96ml
KOSR	15ml	N-2 Supplement (100X) (ThermoFisher, cat# 17502048)	1ml	B-27™ Supplement (50X) with Vitamin A (ThermoFisher, cat# 17504001)	2ml
NEAA	1ml	Pen/Strep	1ml	GlutaMax	1ml
Pen/Strep	1ml			Pen/Strep	1ml
50Mm β-mercaptoethanol	20µl				

Medium was changed daily from Day 0 to Day 20:

On day 0 cells were fed with medium N1 supplemented with 10 μM SB431542 (SB; Miltenyi, cat# 130-105-336) and 100 nM LDN-193189 (LDN; Miltenyi, cat# 130-103-925).

On day 1 and 2 cells were fed with medium N1 supplemented with 10 μM SB, 100 nM LDN, 0.25 μM Smoothened agonist (SAG; Calbiochem, cat# 566660-1MG), 2 μM purmorphamine (Pu; Miltenyi, cat# 130-104-465), and 50 ng/ml fibroblast growth factor 8b(FGF8b; Miltenyi, cat# 130-095-731).

On day 3 and 4 cells were fed with medium N1 supplemented with 10 μM SB, 100 nM LDN, 0.25 μM SAG, 2 μM Pu, 50 ng/ml FGF8b, and 3 μM CHIR99021 (CH; Miltenyi, cat# 130-103-926).

On day 5 and 6 cells were fed with medium of 75% N1 and 25% N2 supplemented with 100 nM LDN, 0.25 μM SAG, 2 μM Pu, 50 ng/ml FGF8b, and 3 μM CH.

On day 7 and 8 cells were fed with medium of 50% N1 and 50% N2 supplemented with 100 nM LDN and 3 μM CH.

On day 9 were fed with medium of 25% N1 and 75% N2 supplemented with 100 nM LDN and 3 μM CH.

On day 10 cells were washed once with PBS w/o Calcium and Magnesium and then detached with Accutase (ThermoFisher, cat# A1110501) (e.g. 1 ml per well of a 6-well plate). Accutase was added at room temperature and cells were incubated for 5 min at 37 °C in the incubator. After the incubation, the plate was tapped until cells started detaching. Cells were diluted 10x with PBS and transferred to a centrifuge tube and spun for 3 min at 300 g. After that, cells were resuspended in day 10 medium (identical to day 9 medium) supplemented with 10 µM Rock-Inhibitor and replated onto a freshly coated Matrigel dish in ratio 1:1.

On day 11 and 12 cells were fed with medium NB-B27 supplemented with 3 μM CH, 10 ng/ml brain derived growth factor (BDNF; Miltenyi, cat# 130-096-285), 10 ng/ml glial derived growth factor (GDNF; Miltenyi, cat# 130-096-290), 1 ng/ml tumor growth factor 3 (TGF3; Miltenyi, cat# 130-094-007), 0.2 mM ascorbic acid (AA; Sigma Aldrich, cat# A7506-25 G) and 0.1 mM cAMP (Enzo Life Sciences, cat# BML-CN125-0100).

From day 13 to day 20 cells were fed daily with medium NB-B27 supplemented with 10 ng/ml BDNF, 10 ng/ml GDNF, 1 ng/ml TGF3, 0.2 mM AA and 0.1 mM cAMP.

At day 20 cells were detached and centrifuged as described for day 10. After that, cells were resuspended in day 20 medium supplemented with 10 µM Rock-Inhibitor and replated onto a freshly coated Matrigel dishes at a density of 250,000 cells/cm^2^. Cells were kept in these wells for terminal maturation and fed with day 20 medium every other day. Once a week, medium was supplemented with 10 µg/ml Laminin (Sigma Aldrich, cat# 11243217001).

### 2.8 Gene expression analysis

For gene expression analysis, RNA was extracted with the RNeasy Mini Kit (Qiagen, cat# 74104) or with RNeasy Plus Micro Kit (Qiagen, cat# 74034) for the RNA extraction from FACS sorted cells, quantified with Nanodrop and RNA integrity was confirmed using Experion™ Automated Electrophoresis System (Biorad, cat#7007103). RNA was retrotranscribed with SuperScript VILO cDNA Synthesis Kit (Thermo Fisher, cat# 11754050). For the quantification of pluripotency genes (Sox2, Oct3/4, nanog, Gdf3) and for TH expression levels during differentiation 5 ng of cDNA were used. cDNA was amplified using All-in-OneSYBR® Green qPCR Mix (GeneCopoeia) on CFX96 Real-Time PCR Detection System (BioRad). ddPCR was carried out using 2 ng of cDNA (corresponding to RNA quantity). For detection of eGFP (FAM; ThermoFisher, cat# Mr04097229_mr), TH (HEX; BioRad, cat# dHsaCPE5192176) and b-actin (FAM; ThermoFisher, cat# Hs01060665_g1) hydrolysis probe based gene expression assays were used. For reaction setup, ddPCR Supermix for Probes (Biorad) was used. Reaction was performed according to standard procedures suggested by manufacturer.

### 2.9 Immunocytochemistry and confocal imaging

Immunocytochemistry was performed on cells at different points of differentiation. On day 25or day 35 in vitro, cells were fixed for co-staining of TH and eGFP. For fixing, cells were washed carefully once with PBS w/o Calcium and Magnesium (ThermoFisher, cat# 10010015) and then fixed with 4% Paraformaldehyde (Sigma Aldrich, cat# P6148) for 15 min at RT. After fixing, cells were washed three times with PBS.

Cells were permeabilized for 5 min at RT in PBS with 0.5% Triton (Sigma Aldrich, cat# T8787) on a shaker. Then, cells were blocked in PBS + 5% donkey serum (Sigma Aldrich, cat# D9663) for 1 h at RT on a shaker. After blocking, fixed cells were incubated in primary antibody solution (antibodies diluted in PBS + 2% donkey serum + 0.2% Triton). See Table 2 for detailed antibodies combination used.

**Table 2:** Antibody combinations and dilutions used for co-staining.

Epitope	Host	Dilution	
α TH	mouse	1:500	(Millipore, cat# MAB318)
α eGFP	rabbit	1:1000	(ThermoFisher, cat# A11122)
α MAP2	chicken	1:5000	(abcam, cat# ab5392)

The primary antibody incubation was carried out ON at 4 °C on a shaker.

Cells were washed three times with PBS and incubated in secondary antibody solution (PBS + 2% donkey serum + 0.2% Triton) for 3 h at RT. See Table 3 for detailed secondary antibody combinations and dilutions.

**Table 3:** Secondary antibody combinations and dilutions used for respective primary antibody.

Epitope	Host	Fluorophore	Dilution	
α rabbit	donkey	AF 488	1:1000	(ThermoFisher, cat# A-21206)
α mouse	donkey	AF 555	1:1000	(ThermoFisher, cat# A-31570)
α chicken	donkey	AF 647	1:500	(Millipore, cat# AP194SA6)

After the secondary antibody incubation, cells were washed again three times with PBS. In the last PBS wash, NucBlue® Fixed Cell ReadyProbes® Reagent (ThermoFisher, cat# R37606) was added to stain cell nuclei. After washing, cover slips were mounted in mounting medium (Dako, cat# S3023) on a microscope slide and sealed with nail polish.

Images of immunostained hiPSCs were acquired with Leica TCS SP8 laser scanning confocal microscope equipped with a white light laser and accusto-optical beam slitters (AOBS) tuned for NucBlue®, AF488, AF555 and AF647.

Pluripotency markers were detected using Pluripotent Stem Cell 4-Marker Immunocytochemistry Kit (Thermo Fisher Scientific) following the manufacturer's instructions. Briefly, after fixation in 4% PFA, cells were permeabilized, blocked and incubated with primary antibody overnight, followed by incubation with the fluorescent antibody.

### 2.10 Fluorescence – activated cell sorting (FACS)

On day 25 of differentiation, cells were dissociated to a single cell suspension with Accutase as described before and pelleted at 300 g for 3 min. Cells were stained with LIVE/DEAD™ Fixable Near-IR Dead Cell Stain Kit (ThermoFisher, cat# L10119) according to manufacturer's instructions. Then cells were re-suspended in sorting buffer (PBS without Ca^2^^+^+/Mg^2^^+^+, Biowest, cat# L0615; 2.5% Horse Serum, Thermofisher, cat# 16050130; 0.4% Glucose, SigmaAldrich, cat# G7021; 5 mM EDTA, SigmaAldrich, cat# EDS; DNAse I, 50 µg/ml, Qiagen, cat# 15200) and filtered through a 35 µm cell filter (BD Bioscience, cat# 340626) to ensure a single cell suspension. Briefly, cells were first gated based on forward scatter and side scatter plot, doublets and dead cells were excluded and eGFP-positive cells were detected in the green fluorescence channel. Cell sorting was performed by FACS Aria III (BD Bioscience) using a ceramic nozzle of size 100 µm and cells were sorted directly into tubes containing lysis buffer (RLT, Qiagen) at 4 °C.

### 2.11 Quantification and statistical analysis

For ddPCR Data analysis, QuantaSoft Software from Biorad was used. Flow cytometry data was analysed using FACS Diva Software and FlowJo, Version 10.

GraphPad Prism 6 (GraphPad Software, La Jolla, CA) software was used for statistical analysis. One-way ANOVA with Bonferroni's multiple comparison test. Unpaired two-tailed *t*-test with Mann-Whitney test for parametric comparison. D'Agostino Pearson omnibus normality test and Spearman correlation for correlation coefficient. Values are indicated in figures as *p < 0.05, **p < 0.01, ***p < 0.001, ****p < 0.0001.

## 3 Results

### 3.1 Generation of knock-in hiPSC lines with CRISPR/Cas9 technology

We developed a CRISPR/Cas9 strategy to engineer three different hiPSC lines (802#7, 856-03-04 and 840-03-05) at the TH locus. First, we designed four different single guide RNAs (sgRNAs) in silico using the online CRISPR Design Tool (http://crispr.mit.edu/; (Ran et al., 2013)). We chose sgRNAs targeting sequences near the stop codon of the TH gene and based on their inverse likelihood for creating off-target mutations. Specifically, the NGG triplets near the stop codon of the TH gene were targeted for the sequence design. To single out one sgRNA, the efficiency of each candidate was tested in vitro using HEK 293T cells and performing a T7 Endonuclease assay (Fig. S1A). In order to faithfully mark TH expression, we constructed a donor plasmid to engineer the TH locus at the stop codon. We aimed to obtain a fluorescent protein expression system under the control of the endogenous promoter. To this purpose, the donor plasmid contained the following insert sequence: a T2A peptide sequence allowing ribosome skipping after TH translation, the eGFP coding gene and a Blasticidine resistance cassette with EF1a core promoter sequence flanked by loxP sites. The insert sequence was flanked by two human TH gene homology arms of approximately 800 bp in length (Fig. 1A). Importantly, the stop codon was eliminated from the TH gene homologous sequence in order to get uninterrupted expression of TH, T2A and eGFP. We electroporated hiPSCs with the donor plasmid, together with an expression vector carrying an enhanced version of S.pyogenes Cas9 and the chimeric sgRNA (Addgene plasmid PX459 V2.0; # 62988). We obtained single hiPSC clones by plating cells at low density on mouse embryonic fibroblast (MEF) feeder cells (see Methods). After one week, single clones were picked up and transferred to a Matrigel coated 96-well plate and amplified in order to obtain three replica plates (Fig. S2A).

### 3.2 Screening of recombination events using ddPCR

To identify successful homologous recombination (HDR) events, we developed a novel ddPCR assay that allows screening of the transfected cell pool as early as four days after electroporation. The assay consisted of two primer-probe sets, where primers were identical for both sets but probes had different sequences and were both marked with FAM fluorophore. The forward primer was set upstream from the starting point of the homology arm region to avoid detection of signal from leftover plasmid after cell electroporation. The reverse primer was designed on the T2A peptide sequence (Fig 1B). Therefore, only on-target HDR events could be detected with this ddPCR strategy. We optimized annealing and extension temperatures using gBlock® fragments, containing the sequence of the target amplicon, that were spiked in with wild-type (WT) genomic DNA. Reactions were multiplexed with a HEX probe for RPP30 (Ribonuclease P protein subunit p30 Ribonuclease P protein subunit p30), which was used as a housekeeping gene in order to be able to quantitate the abundance of the HDR sequence. The optimized ddPCR allowed a quantitative readout of engineering efficiency with a high sensitivity four days after transfection (Fig. 1C). We detected recombination events in all three cell lines and were able to detect events as rare in abundance as 0.37% (Fig. S1B). We quantified an absolute difference of efficiency in three cell lines. HDR efficiency four days after electroporation was of 0.37%, 0.64% and 0.66% for the hiPSC lines 802#7, 856-03-04 and 840-03-05, respectively.

### 3.3 Clonal selection and characterization

After identification of positive HDR events in the cell pool, we selected single hiPSC clones by plating the cell pool at a low density on a MEF feeder layer. After one week of culture on MEF feeder cells, single hiPSC clones were hand-picked and transferred to a Matrigel coated 96-well plate. The 96-well plates were expanded and triplicated in order to obtain three identical replica plates (Fig. S2A). One replica plate was used for genomic DNA extraction and ddPCR screening of single clones. We pooled together columns of a 96-well plate in a first run, to reduce the number of ddPCR reactions. Upon positive signal detection, each well of one column was screened individually to identify the positive clone (Fig. S2B). Signal enrichment could be observed from several clones from all cell lines (Fig. 1D) confirming a clonal identity. Successfully identified clones were singled out and then sequenced at the TH locus to confirm the accuracy of the integrated sequence and to confirm the intactness of the non-engineered allele. Eight out of 9 selected clones were correctly engineered with the WT allele remaining unaffected. Only a single clone showed large Indel regions on the WT allele and was discarded. As an additional quality assessment, we performed FISH with a probe for the insert and homology arm sequence. This was in order to validate the correct chromosomal position of the insert and to exclude off-target integrations of the plasmid sequence elsewhere in the genome. We observed specific hybridization near the p-arm telomere of chromosome 11 (Fig. 1E), corresponding with the chromosomal mapping position of the human TH gene in band 11p15.5, genomic coordinates chr11:2,163,928-2,174,080 (GRCh38). Other hybridization signals were not detected confirming the absence of random integrations of the donor plasmid. We then confirmed that control and edited hiPSC lines correctly maintained their pluripotency status. For this, we immunostained hiPSCs for the established pluripotency markers TRA-1-60, OCT3-4, SSEA4, SOX2. We observed comparable immunosignals in WT and TH-eGFP hiPSCs, which display robust expression of all markers (Fig. S3). To further confirm the pluripotency status of cells, we quantified gene expression of Sox2, Oct3/4, Nanog and Gdf3 via qRT-PCR. The mRNA levels of these markers were undetectable in control fibroblasts and were potently elevated in WT and edited hiPSC lines (Fig. S3).

### 3.4 TH and eGFP are co-expressed and endogenous eGFP is detectable by flow cytometry

**Fig. 2:**
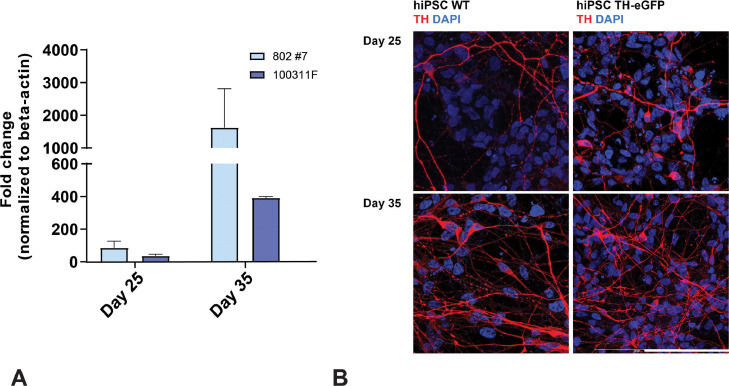
Differentiation of hiPSCs into DA neurons. (A) Time course of TH gene expression in neurons derived from WT hiPSCs (clone 802#7) and TH-eGFP reporter cells (clone 100311F) at in vitro day 25 and day 35. Data shown as fold increase over beta-actin normalized to hiPSCs (mean ± SEM). (B) Immunostaining of TH in WT hiPSCs and in TH-eGFP reporter line at two different time points. TH staining is compared between WT hiPSCs and TH-eGFP hiPSCs at in vitro day 25 and day 35 (scale bar = 100 µm).

**Fig. 3:**
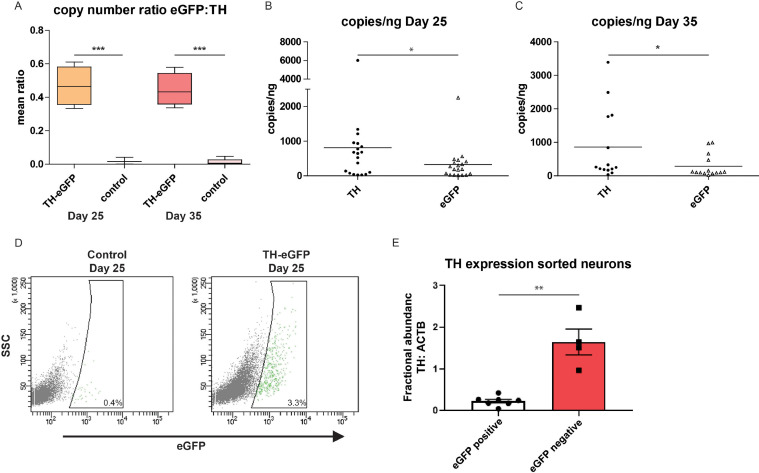
TH and eGFP co-expression. (A) Box whiskers plot showing ratio of expression profile of eGFP and TH at day 25 in vitro and day 35 in vitro. TH-eGFP knock-in clones are compared to WT cell clones. Gene expression was measured with ddPCR. Mean values from 4 different eGFP clones and two WT clones from 5 independent differentiations are shown. (B) Scatter plot showing copies/ng of cDNA detected with ddPCR for eGFP and TH at day 25 in vitro. Values are single data points from 4 different eGFP clones and two WT clones from 5 independent differentiations (mean ± SEM). (C) Scatter plot showing copies/ng of cDNA detected with ddPCR for eGFP and TH at day 35 in vitro. Values are single data points from 4 different eGFP clones and two WT clones from 5 independent differentiations (mean ± SEM). (D) Representative FACS plots of TH-eGFP neurons and control cell line neurons at day 25. Control cell line neurons were used as negative control to draw gates. (E) Plot showing ratio of expression profile of TH of FACS sorted cells from TH-eGFP reporter neurons, FACS-sorted eGFP-positive and eGFP-negative neurons. TH gene expression was measured with ddPCR, normalized to b-actin and expressed as fractional abundance. Mean values from *n *= 3.

In order to detect eGFP expression under the control of the TH promoter, we adapted an efficient differentiation protocol for DA neurons from hiPSCs (Zhang et al., 2014). We measured TH gene expression in WT cells using qRT-PCR to assess the earliest time point at which TH-eGFP co-expression could be detectable and to compare mRNA levels between WT and TH-eGFP cells. We observed a considerable increase in TH gene expression at day 25 in vitro (Fig. 2A), consistent with previous observations (Fedele et al., 2017). In addition, TH gene expression did not quantitively differ between WT and edited neurons (Fig. 2A). Differentiated WT and TH-eGFP neurons were also immunostained for TH to confirm a similar differentiation capacity (Fig. 2B). Next, we tested and directly compared the parallel expression of TH and eGFP in differentiated knock-in reporter lines into DA neurons and extracted RNA at two different time points, day 25 and day 35, when we (Fig. 2A) and others (Adil et al., 2017) show initial (day 25) and more robust (day 35) TH expression. After cDNA production by retrotranscription, we ran ddPCR with two custom assays for TH and eGFP (see Key resource table) to identify copy numbers of cDNA for both genes. Using QuantaSoft software we could determine a ratio of eGFP copy number versus TH copy number at day 25 and at day 35 (Fig. 3A) in TH-eGFP clones. Correctly, we did not detect any eGFP mRNA in non-engineered cells. In addition, the absolute values of cDNA copies/ng for eGFP resulted half that of TH (Fig. 3B), consistent with successful gene editing on a single allele. A significant correlation between TH and eGFP copy number/clone was determined (Spearman correlation coefficient = 0.952, *P*<0.0001, *n *= 33). We were thus able to quantitatively confirm the heterozygous integration of eGFP gene under TH promoter control, confirming observations with the FISH assay, and proving that genetically the reporter system is working accurately.

**Fig. 4:**
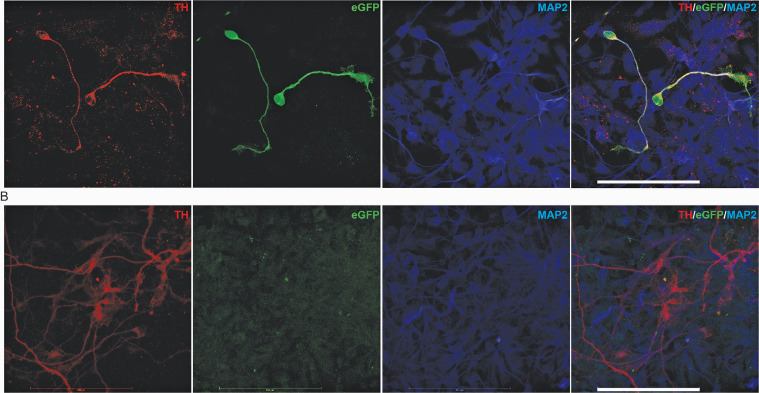
TH and eGFP immunostaining. (A) Co-immunostaining of TH, eGFP and MAP-2 in TH-eGFP knock-in (clone 100311F) neurons at day 25 in vitro (scale bar = 100 µm). (B) Co-immunostaining of TH, eGFP and MAP-2 in WT (line 802#7) neurons at day 25 in vitro (scale bar = 100 µm).

At day 25, we detached cells for fluorescence – activated cell sorting (FACS) in order to isolate TH-eGFP reporter cells. Non-engineered 802#7 cells served as a negative control to draw gates in the FITC channel. When analysing TH-eGFP reporter cells, we detected a fraction of cells that could be clearly distinguished from the non-fluorescent population of cells. In dot plots representing SSC-A against green fluorescence (FITC), the percentage of fluorescent cells was quantified as 3.3% (Fig. 3D). We then analysed TH mRNA expression levels in FACS-purified eGFP-positive and eGFP-negative cells at day 25 of differentiation (a mid-stage of DA neuron induction (Kriks et al., 2011)). ddPCR analysis demonstrated an approximately 10-fold enrichment of TH mRNA levels in eGFP-positive compared to eGFP-negative sorted cells confirming that eGFP fluorescence identifies DA neurons within a heterogenous population and our approach is specific in editing TH-expressing DA neurons (Fig. 3E). To confirm the co-presence of TH and eGFP, we then performed immunostaining for both proteins and subsequent confocal imaging in TH-eGFP reporter cells. In line with the combination of FACS and ddPCR TH gene expression, we observed co-expression of TH and eGFP in differentiated DA neurons in TH-eGFP reporter cells (Fig. 4). We detected eGFP signal at day 25 (Fig. 4A) in neurons that were also positively stained for TH and for the neuronal marker MAP2. The signal for eGFP was absent in neurons differentiated from non-engineered parental hiPSCs (Fig. 4B). We were not able to detect endogenous eGFP fluorescence on fixed cells. In addition, we determined that 82% of TH positive cells were also co-expressing eGFP, underlining the specificity of the TH-driven expression of the fluorescent tag (3 independent differentiations, 10 image fields per differentiation; data not shown).

These data show that not only does the reporter mimic the expression on mRNA but also on protein level. Flow cytometry and FACS enable quantitative analysis of the cell population at a single cell resolution and allows a greater sensitivity in the detection of endogenous eGFP fluorescence signal.

## 4 Discussion

In this study, we report a successful strategy for the generation of a TH-driven fluorescent reporter for DAergic neurons using CRISPR/Cas9 genome engineering in combination with ddPCR for HDR screening. We show that this approach results in a faithful co-expression of TH and an eGFP reporter at the single cell level. Specifically, we successfully integrated a 1500 bp-long fragment into control hiPSC lines by providing a plasmid-based donor sequence flanked by 800 bp homologous sequences. A seamless integration was generated without disrupting the open reading frame of the genes involved, producing hiPSCs expressing a fluorescent protein tag under the control of an endogenously expressed gene, thereby creating reporter lines for DA neurons. This aspect is particularly relevant, as it allows targeting and identification of this specific neuronal cell type characterized by a distinct temporal expression pattern during differentiation. We chose an engineering strategy that forgoes the creation of a fusion protein. In vitro differentiation is a highly complex biological process and the risk of disrupting this process by creating a fusion protein for one of the molecular hallmarks of DA neurons with no foreseeable impact of such a product could not be undertaken. In addition, a fusion protein would generate a hybrid TH protein, with the possibility of altering its function and/or subcellular trafficking. Therefore we opted for a strategy that allowed the co-expression and co-translation (Wang et al., 2015) of both proteins independently in order to guarantee their physiological functionality. We show here that this construct leads to a perfect overlap of gene expression. Also, a co-presence of both proteins could be observed in the same neuron. To facilitate the screening of engineered hiPSCs, we designed a ddPCR method, which was validated not only to detect the insertion of the fluorescent tag but also to determine the correct locus of recombination. In fact, even though advancements in CRISPR/Cas9 technology led to a higher level of recombinant accuracy by enhanced variants of Cas9 (Kato-Inui et al., 2018; Slaymaker et al., 2016), we could not exclude random plasmid integration causing expression of the fluorescent tag in a TH promoter-independent manner. We designed a ddPCR assay spanning the targeted genomic region upstream of the homology sequence cloned and fused with the fluorescent tag. Therefore, only after integration at the correct genomic locus, the PCR product could be amplified allowing identification of positively engineered clones. We optimized this technology in order to identify even very low-abundance events of successful homologous recombination. Introducing this screening as early as four days after transfection, allowed us to estimate the number of clones to be selected by hand, and to discard unsuccessfully transfected cell lines. Moreover, through the ddPCR screening method we were able to determine the absolute value of successfully engineered cells. This clearly indicated that differences in engineering efficiency exist and that they are cell line-dependent, an effect that has yet not been described, but can clearly be reported in a quantitative manner with our method. Most important is that applying this screening method for genome engineering experiments allows us to perform a clone screening a priori, before continuing with the manual selection of the hiPSC clones, which is cost and work intensive and should be avoided if possible. The co-expression of TH and eGFP is confirmation of successful genetic engineering, and we addressed this using complementary methodologies. First, we demonstrated using ddPCR that mRNA expression of both the target gene (TH) and the reporter (eGFP) is perfectly aligned on the basis of the homologous recombination on a single TH allele, with eGFP mRNA being half that of TH. This proved that, at the genomic level, the tandem of TH-T2A-eGFP is fully functional and that the eGFP expression profile mimics that of TH, thus enabling the potential selective identification of DA neurons upon differentiation. Our ddPCR gene expression approach first describes a method to quantify gene load of such a gene-T2A-reporter tandem in order to validate its functionality. We were not able to detect endogenous fluorescence for eGFP through confocal imaging. In our study, the expression of the reporter tag is under the control of an endogenously regulated gene, therefore quite different from vector-based recombination systems with expression of a gene under the control of a viral promoter (e.g. CMV) with high abundance of the fluorescent tag. In fact, in our study the signal-to-noise ratio between reporter tag-driven fluorescence versus autofluorescence is reduced thus impairing the detection of the fluorescent signal even by confocal microscopy. To overcome background fluorescence, eGFP has to be at a concentration of at least 200 nM, a protein load two times that of MAPKKK (Snapp, 2005). An impairment in translation using a T2A system is also possible to some extent (Wang et al., 2015), that may reduce the protein load for eGFP further, thus minimizing the brightness of an endogenous signal. However, we were able to detect the fluorescent proteins with indirect labelling in immunocytochemistry experiments. An overlapping TH-eGFP expression was observed upon differentiation into DA neurons thus confirming the gene expression data and the functionality of the TH-T2A-eGFP tandem construct. The fact that endogenous eGFP fluorescence is detectable in flow cytometry is indeed pivotal, confirming that the detection of the endogenous fluorescence is a technical matter of instrument sensitivity and background fluorescence compensation. Furthermore, it demonstrates that the created knock-in faithfully reports TH expression in vitro. This is notable, as previous approaches to a TH-reporter system failed to deliver faithful co-presence of both proteins (Kelly et al., 2006) or were not based on endogenous gene expression (Hedlund et al., 2007). A study was published in 2017 (Xia et al., 2017) with a similar construct for DA neurons to that reported here, using a red fluorescent protein, demonstrating the feasibility of using this kind of reporter system. Compared to this previous study (Xia et al., 2017), we detect an increased proportion of TH positive cells with our eGFP reporter, which potentially represents a more suitable fluorophore for flow cytometry experiments requiring a high sensitivity such as eGFP. Indeed we demonstrate that our system is suitable for sorting enriched TH-expressing neurons based on the (endogenous) fluorescence signal emitted by the reporter, as proven by the increased number of TH mRNA copies in eGFP-positive FACS-sorted neurons, compared to eGFP-negative ones, in an extent that matches the percentage of eGFP positive sorted cells. Thus, our approach allows the specific enrichment of the DA neuronal cell population for molecular studies, with the potential to sort viable TH-expressing neurons while maintaining the integrity of the cell membrane for functional investigations. At the same time, it delivers the most important application of this kind of reporter construct. Being intended to distinguish DA neurons from non-DA neurons, we can in this manner not only discriminate a certain population but also separate it to enrich the DA neuronal cell population in culture. Most importantly, our approach can be applied also to cells derived from mutation-carrying patients in order to isolate patient or healthy control DA neurons in parallel and directly compare phenotypes from the cell type of interest without suffering contaminations from non-DA cells. As there is no widely accepted surface marker for DA neurons (Lehnen et al., 2017), the availability of an endogenous fluorescent reporter is a major step forward to producing a more homogeneous TH-positive neuronal culture. In conclusion, our results demonstrate that we have developed a reproducible method that facilitates the creation of knock-in cell lines in hiPSCs. We developed this method on a DA neuron specific locus but it is applicable to any other desired locus throughout the genome. The ddPCR method is a technique with increasing potential to facilitate genome-editing experiments (Miyaoka et al., 2018) and we show that its application to clonal screening offers considerable advantages in mutation detection because of its high sensitivity. Additionally, here we have shown that TH-eGFP reporter system could represent a reliable method to address DA neuron physiology, their differentiation, and their connection to disease development. The potential to sort these cells as neuronal DAergic precursors could greatly contribute to the development of regenerative medicine therapeutic approaches in PD.

## Declaration of Competing Interest

The authors declare that they have no known competing financial interests or personal relationships that could have appeared to influence the work reported in this paper.

## Appendix Supplementary materials

Image, application 1
