# Supplementary material for: Application of CRISPR/Cas9 editing and digital droplet PCR in human iPSCs to generate novel knock-in reporter lines to visualize dopaminergic neurons
Source: Stem Cell Res. 2019 Dec;41:101656. doi: 10.1016/j.scr.2019.101656 (PMC7322529; doi:10.1016/j.scr.2019.101656)
Supplement: Supplementary file 1 [file mmc1.zip › mmc1/berbacher 2018_StemCellResearch_Supplementary Information.docx]

Supplementary procedures

1. Clonal Selection of engineered clones
   1. Clonal plating of reporter cells

5 days after electroporation, puromycin-selected cells were plated at low density on mitotically inactivated, irradiated MEF feeder cells (Millipore, cat# MEF-CFL) (10,000 iPSCs on 300,000 MEF cells per 10cm^2^) and fed with stem cell medium (Knock-out DMEM, ThermoFisher,cat# 10829018; 20% KOSR, ThermoFisher, cat# 10828028; 1% MEM-NEAAs, ThermoFisher, cat# 11140035; 1% Penicillin/Streptomycin, ThermoFisher, cat# 15140122; 1% GlutaMAX^TM^, ThermoFisher, cat# 35050061; 0.1 mM β-mercaptoethanol, ThermoFisher, cat# 31350010; and 10 ng/ml basic FGF (Millipore, cat# GF003). After 7 days isolated colonies were picked manually under a dissecting microscope in a Class 2 cabinet using a P200 pipette tip and each transferred to a separate well of a Matrigel coated 96 well plate. hiPSC clones were cultured until wells were confluent and then split at ratio 1:3 with EDTA in order to obtain three identical 96well plates. One was used for gDNA extraction, two were frozen for backup (see below).

- 1. Passaging and freezing hiPSC clones in 96 well plates

For splitting, a fresh 96 well plate coated with Matrigel was prepared. Medium from 96 well plates with cells was aspirated using the vacuum pump with an 8-channel adaptor. Wells were washed once with PBS. Then, wells were incubated in 50µl of PBS containing 0.5mM EDTA for 5 min at 37°C. After 5 min, EDTA solution was aspirated completely and plate was moved back to incubator for another 5 min. EDTA does not detach the cells from the plate when used for this amount of time and without mechanical disruption, so there was no risk to aspirate them. After the second round of incubation to each well 100µl of warm StemMACS with 10µM Rock-Inhibitor was added. With a multichannel pipette, medium was pipetted up and down gently five times in order to detach cells from wells. 50µl of the cell suspension were transferred to the freshly prepared 96 well plate. The remaining 50µl were left in the original plate.

Cells were incubated further with daily medium change. After 2 days splitting procedure was repeated to obtain 3 identical plates (see Fig S1B). After another 2-3 days of expansion, two of the three wells were frozen as backup plates.

For freezing the 96 well plates, the procedures for splitting were followed until after EDTA incubation. At this point cells were resuspended in 50µl of Freezing medium consisting of 60% Medium, 30% ES-qualified FCS (ThermoFisher, cat# 16141079) and 10% DMSO (Sigma Aldrich, cat# D2650). Cells were again pipetted up and down gently 5 times and then the plate was sealed with an adhesive aluminium foil. To allow freezing with a controlled temperature gradient, the plates were transferred to a polystyrene box filled loosely with paper towels, which was put into a -80°C freezer overnight. The next day, the plate was transferred to the nitrogen vapour tank for long-term storage.

- 1. Genomic DNA Extraction from 96well plate

Lysis Buffer:

10mM Tris pH 7.5

10mM EDTA

10mM NaCl

0.25% Triton X100

1mg/ml Proteinase K (Sigma Aldrich, cat# P6556; add fresh before lysis)

Precipitation Solution:

75mM NaCl in 100% ethanol, store on -80°C

70% ethanol for washing

All the steps were carried out using a multichannel pipet. The 96-well plates were washed once with PBS. To each well, 50µl of lysis buffer containing 1mg/ml Proteinase K was added and the plate was sealed in a plastic bag containing wet tissue papers. The plate was incubated in the bag ON at 55°C in an incubator.

The next day 100µl ice cold (-80°C) precipitation solution was added into the lysis buffer without mixing. Precipitation of DNA was allowed for 2h at RT. After this time, precipitated DNA was visible on the bottom of the well as a white thread. The plate was carefully inverted to discard the buffers, while the gDNA stuck to the plate’s bottom. The plate was washed three times with 70% ethanol, always carefully inverting between washes. Remaining ethanol was aspirated with a P10 and residual ethanol evaporated during an incubation at RT with open lid for 10-15 min. gDNA was resuspended in 50µl H_2_O and incubate 1h at 55°C or ON at 4°C for dissolving DNA completely in H_2_O. Plate was centrifuged at 800g for 1min.

- 1. Clonal screen of 96well plates

For the identification of positive clones, again, ddPCR was used for the detection of the same signal as in the HDR screen, looking for a signal enrichment.

The wells of each column of a plate were pooled (see Fig S1C). If in one of these pooled reactions, a positive signal was detected, all wells of this column were analyzed in a separate ddPCR reaction to identify the single positive wells.

For pooling, 1µl of each well of a column was taken and mixed with 1µl of the other wells of a column. For ddPCR 1µl of this mixture was used.

1. The accession number for homology arm design for TH locus used in the preparation of the donor construct is: NG_008128.1. The sequence of the donor construct is given below in FASTA format. See underlined sequences for T2A, loxP sites, eGFP, core promoter and Blasticidine resistance.

FASTA Sequence of TH Donor

>TH Donor

gcagacctacaggactgggggctgctgggtggccgggtcaaggccagtcttggaggtgctgacagagcctgagctttgtgaggacgtcctgtggaacctgtcccggccccctgccctgggatggggagaagtcagggggatagacagagtcaaggtgggggacagggcgggagtggggtccccagggctgggggcctttggtgcagtgaccagagtgtcaggagaggggagcaaagccctctagcctcatcctcataaaaggtctcatcattttccctccagcctcttatgcactggggaaactgaggccaggggctatgtgtccagcggacaggggtgctgaattccacccacaggcttagggatatggtcaaggaaagcttcctggaggaggcccagtggaggttcagggagggatggggtgcccggcagtctctagtggaaaaggcgcctagcctatctcccccatgaaccccctcacccagccctggaagaggcctcagtgtcccgcctgtgaccagttggctcagaaaagccctgggagctctgagccactgtgaaggtggaaacgcggcccctggcctcccctctcctggaggctgcagactctgcccgccagttgacgagggctctgccgctctcctccccaggagctatgcctcacgcatccagcgccccttctccgtgaagttcgacccgtacacgctggccatcgacgtgctggacagcccccaggccgtgcggcgctccctggagggtgtccaggatgagctggacacccttgcccatgcgctgagtgccattggcTCTAGAGGcagCGGCGAGGGCAGAGGAAGTCTTCTAACATGCGGTGACGTGGAGGAGAATCCCGGCCCTACGCGTATGGTGAGCAAGGGCGAGGAGCTGTTCACCGGGGTGGTGCCCATCCTGGTCGAGCTGGACGGCGACGTAAACGGCCACAAGTTCAGCGTGTCCGGCGAGGGCGAGGGCGATGCCACCTACGGCAAGCTGACCCTGAAGTTCATCTGCACCACCGGCAAGCTGCCCGTGCCCTGGCCCACCCTCGTGACCACCCTGACCTACGGCGTGCAGTGCTTCAGCCGCTACCCCGACCACATGAAGCAGCACGACTTCTTCAAGTCCGCCATGCCCGAAGGCTACGTCCAGGAGCGCACCATCTTCTTCAAGGACGACGGCAACTACAAGACCCGCGCCGAGGTGAAGTTCGAGGGCGACACCCTGGTGAACCGCATCGAGCTGAAGGGCATCGACTTCAAGGAGGACGGCAACATCCTGGGGCACAAGCTGGAGTACAACTACAACAGCCACAACGTCTATATCATGGCCGACAAGCAGAAGAACGGCATCAAGGTGAACTTCAAGATCCGCCACAACATCGAGGACGGCAGCGTGCAGCTCGCCGACCACTACCAGCAGAACACCCCCATCGGCGACGGCCCCGTGCTGCTGCCCGACAACCACTACCTGAGCACCCAGTCCGCCCTGAGCAAAGACCCCAACGAGAAGCGCGATCACATGGTCCTGCTGGAGTTCGTGACCGCCGCCGGGATCACTCTCGGCATGGACGAGCTGTACAAGTAAGTCGACcttaatataacttcgtataatgtatgctatacgaagttaTGGCTCCGGTGCCCGTCAGTGGGCAGAGCGCACATCGCCCACAGTCCCCGAGAAGTTGGGGGGAGGGGTCGGCAATTGAACCGGTGCCTAGAGAAGGTGGCGCGGGGTAAACTGGGAAAGTGATGTCGTGTACTGGCTCCGCCTTTTTCCCGAGGGTGGGGGAGAACCGTATATAAGTGCAGTAGTCGCCGTGAACGTTCTTTTTCGCAACGGGTTTGCCGCCAGAACACAGGTGTCGTGACGCGGGATCCgccaccATGGCCAAGCCTTTGTCTCAAGAAGAATCCACCCTCATTGAAAGAGCAACGGCTACAATCAACAGCATCCCCATCTCTGAAGACTACAGCGTCGCCAGCGCAGCTCTCTCTAGCGACGGCCGCATCTTCACTGGTGTCAATGTATATCATTTTACTGGGGGACCTTGTGCAGAACTCGTGGTGCTGGGCACTGCTGCTGCTGCGGCAGCTGGCAACCTGACTTGTATCGTCGCGATCGGAAATGAGAACAGGGGCATCTTGAGCCCCTGCGGACGGTGCCGACAGGTGCTTCTCGATCTGCATCCTGGGATCAAAGCCATAGTGAAGGACAGTGATGGACAGCCGACGGCAGTTGGGATTCGTGAATTGCTGCCCTCTGGTTATGTGTGGGAGGGCTAAGGGCCCcttaatataacttcgtataatgtatgctatacgaagttattaggtcCTCGAGgtgcacggcgtccctgagggcccttcccaacctcccctggtcctgcactgtcccggagctcaggccctggtgaggggctgggtcccgggtgccccccatgccctccctgctgccaggctcccactgcccctgcacctgcttctcagcgcaacagctgtgtgtgcccgtggtgaggttgtgctgcctgtggtgaggtcctgtcctggctcccagggtcctgggggctgctgcactgccctccgcccttccctgacactgtctgctgccccaatcaccgtcacaataaaagaaactgtggtctctacacctgcctggccccacatctgtgccacagagacagaccctgggatcctcagactcccacacccccaccccagcctcactcagaggtttcgccctggcctccttcctcctctgggagatggctggccgccctggccaggcagctggcccctccgggcctggtttccccgctcaccctgaggccccgcccagctctgagccccaagcagctccagaggctcgggcaccctggccgagctgccccatctccgtggggtgccctcccaaggtggggagccacgtgacagtgggagggcctctctcaggcctggcagggagcaggggtcacaaactgtgctggctgggggtggtctcagaggtgggcctgcaggcctaaccctccctgctgacagggctcccagcccttgagagaaacagggatggaggaacagctgccctgatgccctcacccacccggagcaggccctgcgaaccaaggggaacctcagtgtggccccc
