# Supplementary material for: Application of CRISPR/Cas9 editing and digital droplet PCR in human iPSCs to generate novel knock-in reporter lines to visualize dopaminergic neurons
Source: Stem Cell Res. 2019 Dec;41:101656. doi: 10.1016/j.scr.2019.101656 (PMC7322529; doi:10.1016/j.scr.2019.101656)
Supplement: Supplementary file 1 [file mmc1.zip › mmc1/Supplemental Figure caption.docx]

**Supplemental legends**

**Fig. S1**. Engineering of hiPSC lines with CRISPR/Cas9

(A) Surveyor Endonuclease Assay for testing sgRNAs. Four sgRNAs were tested and load together with a negative control (-cntrl).

(B) Fractional abundance of HDR signal compared to housekeeping gene RPP30 four days after electroporation of all three hiPSC lines. Values are automatically calculated by QuantaSoft software (Biorad).

**Fig. S2**. Work flow for clonal selection of hiPSCs carrying knock-in

(A) Schematic representation of work flow of clone selection after electroporation

(B) Representation of screening strategy of manually selected cell clones with ddPCR

**Fig. S3**. Characterization of pluripotency markers in hiPSCs

(A) Gene expression levels of pluripotency genes

(B) Immunofluorescence staining for pluripotency markers SSEA4, OCT4, SOX2 and TRA-1-60

**Fig. S4**. TH and eGFP immunostaining

Co-immunostaining of TH, eGFP and MAP-2 in TH-eGFP reporter (clone 100311F) neurons at day 25 in vitro (scale bar = 100µm)
