# Supplementary figures and images for: Application of CRISPR/Cas9 editing and digital droplet PCR in human iPSCs to generate novel knock-in reporter lines to visualize dopaminergic neurons
Source: Stem Cell Res. 2019 Dec;41:101656. doi: 10.1016/j.scr.2019.101656 (PMC7322529; doi:10.1016/j.scr.2019.101656)

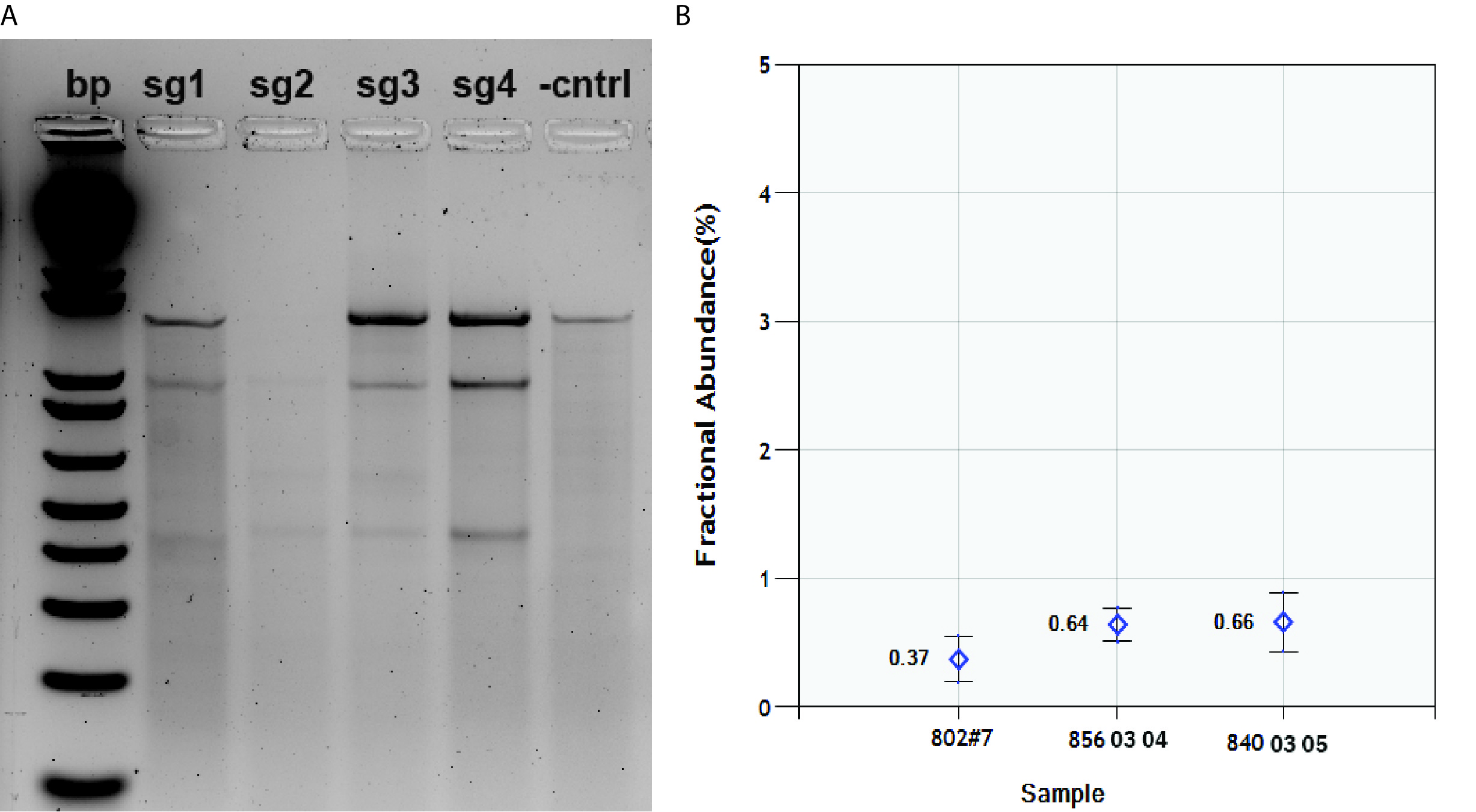

Supplement: Supplementary file 1 [file mmc1.zip › mmc1/Figure S1.jpg]

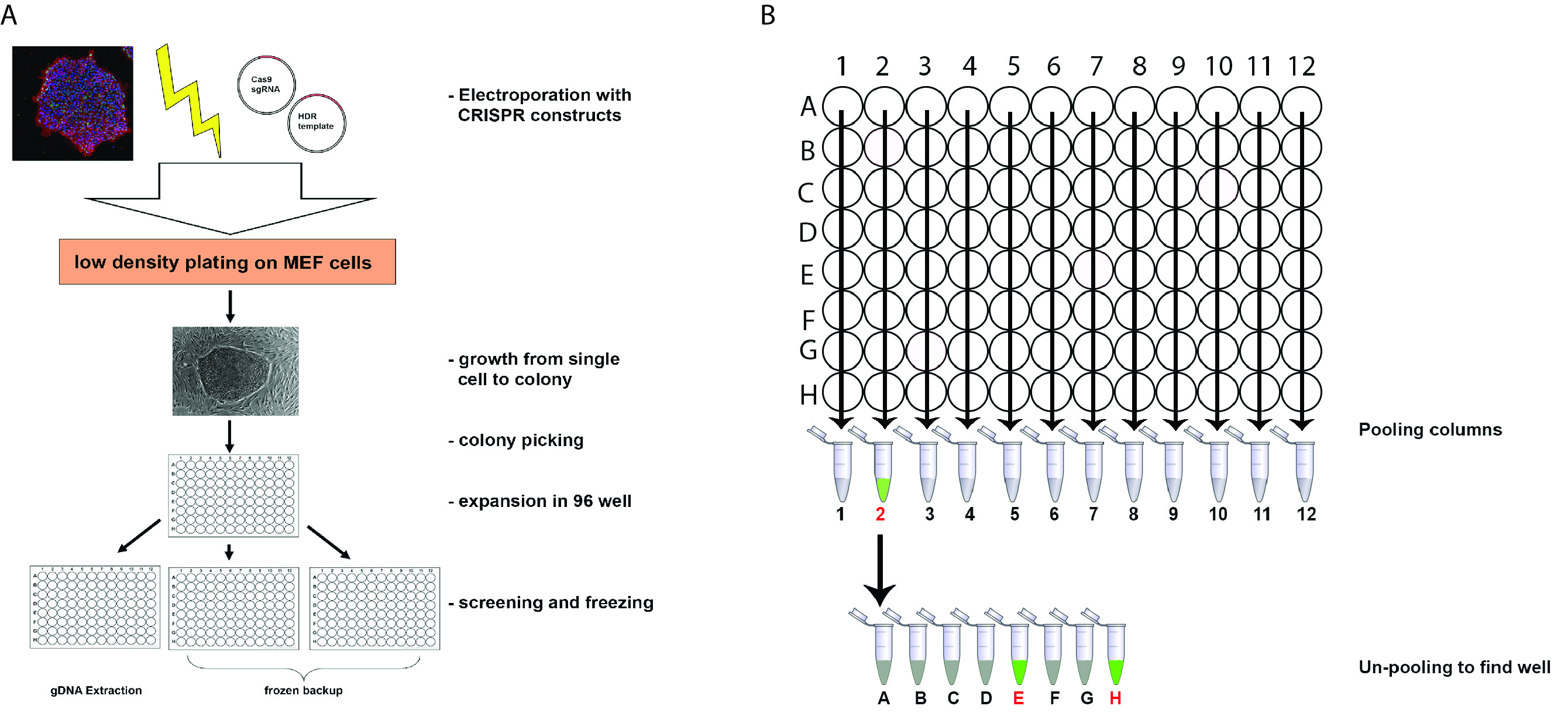

Supplement: Supplementary file 1 [file mmc1.zip › mmc1/Figure S2.jpg]

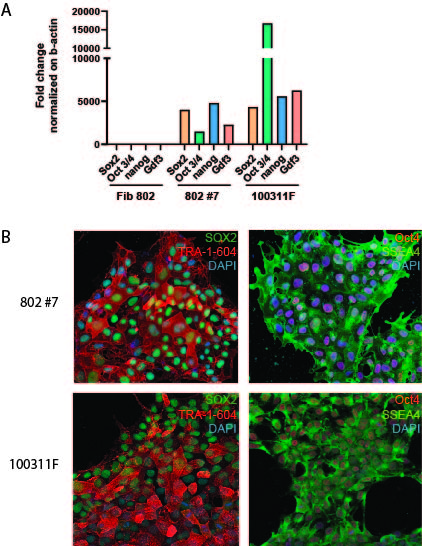

Supplement: Supplementary file 1 [file mmc1.zip › mmc1/Figure S3.jpg]

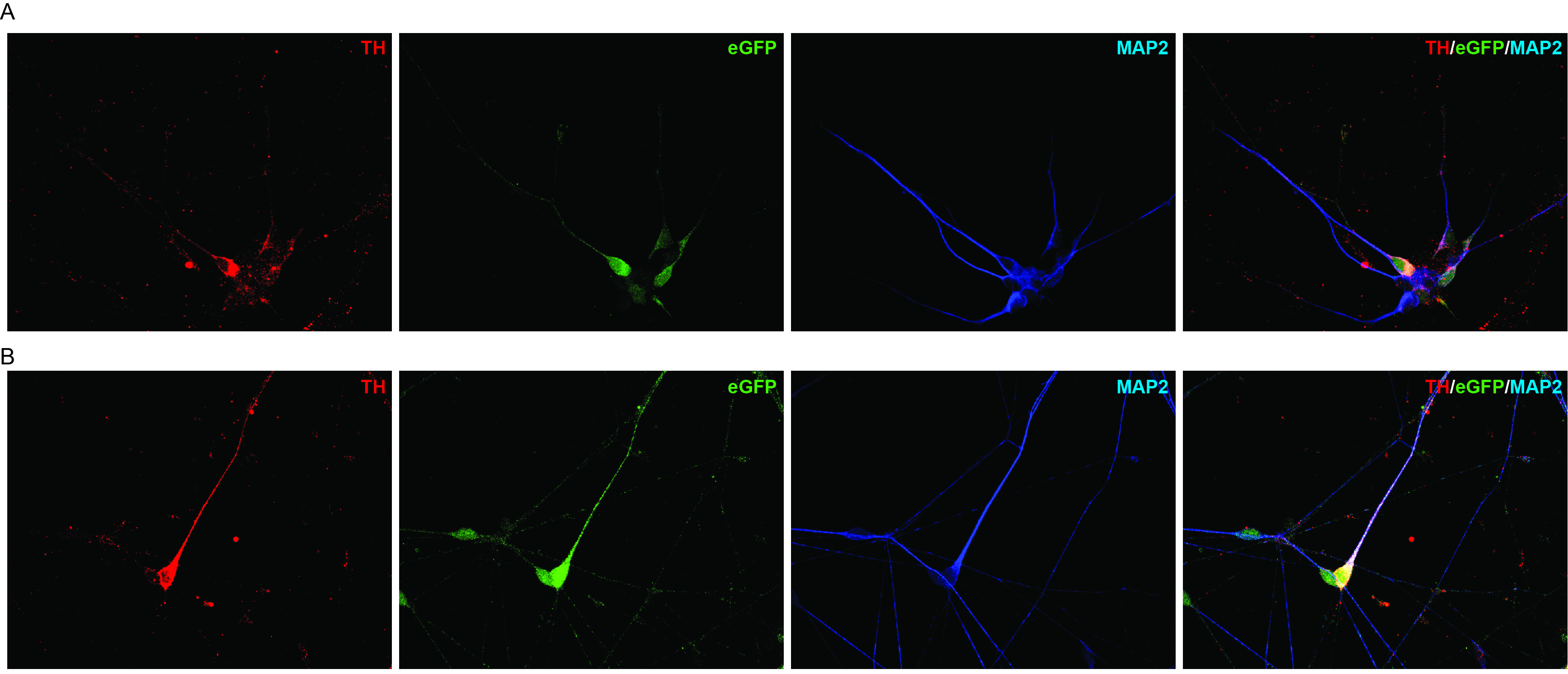

Supplement: Supplementary file 1 [file mmc1.zip › mmc1/Figure S4.jpg]
